# TF-343 Recovered Cyclophosphamide-Induced Compromised Immune Status in a Mouse Model

**DOI:** 10.4014/jmb.2506.06034

**Published:** 2025-10-15

**Authors:** Gun-Dong Kim, Sang Hyuk Yoo, Kyung Min Lim, Ju Hye Song, Seungil Kim, So-Young Lee, Seon-Hee Kim, Hee Soon Shin

**Affiliations:** 1 Research Division of Food Functionality, Korea Food Research Institute (KFRI), Wanju 55365, Republic of Korea; 2 Department of Food Biotechnology, Korea University of Science and Technology (UST), Daejeon 34113, Republic of Korea; 3 Sungkyun Biotech Co., Ltd., Suwon 16648, Republic of Korea

**Keywords:** Cyclophosphamide, immune compromised model, lymphocyte, natural killer cell, macrophage

## Abstract

Cyclophosphamide (CP) exerts potent cytostatic and immunosuppressive effects by inhibiting the proliferation and function of tumor and immune cells, thereby being widely used for therapeutic approaches and molecular mechanism studies in immunocompromised models and autoimmune disease models. TF-343 is a mixed extract derived from medicinal plants with anti-inflammatory and antioxidant properties and exhibits anti-inflammatory activity by modulating signaling pathways associated with immune responses. To elucidate the potential immunomodulatory effects and underlying mechanisms of TF-343 in more detail, we investigated its efficacy in a CP-induced immunosuppressive mouse model. Administered TF-343 significantly recovered CP-induced decreases in body weight and organ indices, including those of the thymus and spleen. The CP-mediated decrease in the number of lymphocytes and white blood cells and immunoglobulin production were restored by TF-343 administration. Furthermore, TF-343 enhanced the proliferation of total splenocytes and their subpopulations, including T and B lymphocytes. The administration of TF-343 promoted the function of natural killer cells and macrophages in CP-induced immunocompromised mice. Additionally, TF-343 supplementation reverted the CP-elicited downregulation of interleukin 2 and interferon γ mRNA and protein expression in splenocytes. Therefore, our results demonstrate that TF-343 enhances immune function and has potential as an immunoregulatory substance.

## Introduction

The immune system is a highly coordinated and elaborated network of cells, tissues, and organs that is the first and most critical line of defense against a wide range of pathogens, including bacteria, viruses, fungi, and other harmful agents [1]. The immune system is broadly categorized into innate and adaptive immunity, which is essential for maintaining immune homeostasis and orchestrating effective defense mechanisms against pathogenic insults [2]. Innate immunity provides an immediate, non-specific response through the phagocytic cells, such as macrophages, neutrophils, and natural killer (NK) cells, which exert cytotoxic functions and produce immunomodulatory cytokines such as interferon γ (IFNγ) and tumor necrosis factor (TNF) [3]. Macrophages, a pivotal component of innate immunity, contribute to engulfing and eliminating pathogens and secrete pro-inflammatory cytokines, including interleukin (IL) 6, IL1β, TNF, and nitric oxide, all of which orchestrate immune signaling through major pathways such as mitogen-activated protein kinases and nuclear factor κB (NFκB) [4]. Adaptive immunity, which is characterized by antigen specificity and immunological memory, involves the activation and clonal expansion of B and T lymphocytes [5]. B cells mediate humoral responses by producing antigen-specific antibodies upon activation, whereas T cells, including CD4^+^ helper T cells and CD8^+^ cytotoxic T cells, govern cell-mediated immunity [6, 7]. T cells secrete cytokines such as IL2, IL6, and IFNγ that facilitate the activation of B cells and other immune components [8-10]. These complex immunological interactions ensure a rapid, specific, and sophisticated regulated response to external antigens while preserving self-tolerance and immunological memory.

Cyclophosphamide (CP), a bifunctional alkylating agent, is extensively used in clinical oncology and immunology owing to its potent immunosuppressive and cytotoxic properties [11]. Clinically, CP is routinely employed to induce immune tolerance during organ and bone marrow transplantation and to treat severe autoimmune diseases such as systemic lupus erythematosus and vasculitis, and to manage diverse malignancies [12]. Mechanistically, CP is a prodrug requiring hepatic activation into alkylating metabolites that cross-link DNA, thereby disrupting replication and inducing apoptosis [13, 14]. This process preferentially affects rapidly proliferating cells, including lymphocytes, conferring profound immunosuppressive effects through the depletion of immune cell subsets and modulation of regulatory T cells and dendritic cell homeostasis [15, 16]. Despite its therapeutic efficacy, CP exerts considerable off-target toxicity, including hepatotoxicity and hematologic disorders such as anemia, leukopenia, alopecia, and gastrointestinal mucosal damage, all of which significantly limit its long-term clinical use [11, 17]. These systemic toxicities necessitate careful dosing regimens, prophylactic co-medications, and vigilant clinical monitoring to optimize its therapeutic index [18]. Additionally, CP exerts the capacity to induce immunosuppression has been extensively harnessed to establish immunocompromised animal models, providing valuable platforms for investigating novel immunomodulatory agents and for developing strategies to mitigate its inherent toxicities [19, 20].

Medicinal herbs, widely used in traditional remedies, are increasingly recognized as potent immunomodulatory agents with multifaceted biological activities [21]. Unlike synthetic drugs, which often carry significant side effects and the risk of resistance, medicinal herbs generally exhibit a more favorable safety profile [22]. Traditional herbal formulations typically comprise multiple botanicals prescribed in combination, based on established principles of synergy and balance rather than the action of individual components. These mixed medicinal herb preparations enhance therapeutic efficacy through synergistic interactions and mitigate adverse effects by counterbalancing the toxicity of the individual constituents [23].

TF-343 is a multi-herbal extract of eight medicinal plants, *Adenophora triphylla*, *Cassia tora*, *Glycine max*, *Glycyrrhiza uralensis*, *Lonicera japonica*, *Saururus chinensis*, *Taraxacum platycarpum*, and *Ulmus macrocarpa*, which contain various polyphenolic and flavonoid constituents, such as luteolin, rutin, chlorogenic acid, quercetin, kaempferol, and caffeic acid, which have antioxidative and anti-inflammatory properties [24]. Previous studies have demonstrated that TF-343 mitigates particulate matter-induced lung inflammation and intracellular reactive oxygen species levels by modulating NFκB, a critical regulator of immune signaling pathways associated with inflammation and oxidative stress [24]. However, the immunomodulatory capacity of TF-343 has not yet been investigated. Therefore, we established a CP-induced immunosuppressed mouse model, a widely recognized platform for evaluating the immunomodulatory effects of natural compounds, to assess the immunoregulatory effects of TF-343.

## Materials and Methods

### Plant Materials and TF-343 Preparation

TF-343 is a mixed herbal extract from eight medicinal plants, *A. triphylla*, *C. tora*, *G. max*, *G. uralensis*, *L. japonica*, *S. chinensis*, *T. platycarpum*, and *Ulmus macrocarpa*. These medicinal herbs were purchased from the Kyungdong Market (Republic of Korea) and extracted with hot water in a 100-l extractor (TAEIN F&C Co., Republic of Korea). The filtered extract was powdered using a spray dryer, subsequently freeze-dried (TF-343), and then stored at -80°C until use.

### Animal Experiment

Five-week-old male BALB/c mice (18–22 g) were purchased from Orient Bio Inc. (Republic of Korea), acclimated for one week, and maintained under specific-pathogen-free conditions with a temperature of 23 ± 2°C, 50 ± 5%relative humidity, and a regular 12 h light/dark cycle. Mice were randomly divided into five groups (n =5): negative control, CP injection (CP), 200 mg/kg red ginseng (RG) supplementation with CP injection (positive control), and 200 mg/kg and 400 mg/kg TF-343 supplementation with CP injection (TF-343^low^ and TF-343^high^). To establish an immunocompromised model, 100 mg/kg CP was injected intraperitoneally on day 14. Oral administration was performed daily from day 1 to 17; the negative control group received 200 μl of PBS, while the TF-343 groups received either 200 mg/kg or 400 mg/kg TF-343 resuspended in 200 μl of PBS. RG (Rg1, Rb1, and Rg3 total ginsenosides contents was 16.5 mg/g) was kindly provided by Huons Foodience Co., Ltd. (Republic of Korea) and administered as a positive control. The mice were euthanized using isoflurane inhalation on day 17, and their blood and organs were collected for subsequent studies. All animal procedures were approved by and performed in accordance with the Institutional Animal Care and Use Committee guidelines of the Korea Food Research Institute (approval number: KFRI-M-24048). Harvested total blood samples were subjected to hematopoietic compartment analysis using an automatic blood cell analyzer (ADVIA 2120i; Siemens Healthcare Diagnostics, Germany).

### Splenocyte Proliferation Assay

A water-soluble tetrazolium 1 (WST-1) assay was used to assess the proliferative effects of TF-343 on splenocytes. The spleen extracted from immunocompromised mice was homogenized and removed erythrocytes, then isolated splenocytes were cultured in 24-well plates at a density of 5 × 10^6^ cells/well with RPMI 1640 medium containing 10% fetal bovine serum and 1% antibiotics and treated with 1 μg/ml concanavalin A or lipopolysaccharide. Splenocytes were then incubated at 37°C for 48 h in a humidified atmosphere of 5% CO_2_ and 95% air. After incubation, splenocytes were centrifuged at 1,500 rpm for 5 min to adhere to the bottom of the cell culture plate, and the WST-1 solution was mixed with the medium at a 1:10 ratio. Then, splenocytes were incubated at 37°C for 30 min. The absorbance of each well was measured at 450 nm using an Epoch microplate reader (BioTek, USA).

### Splenic NK Cell Cytotoxicity Assessment

Splenocytes isolated from immunocompromised mice were co-cultured with 4 × 10^4^ cells/well of YAC-1 cells (ATCC, USA) for 4 h at 37°C in a humidified atmosphere of 5% CO_2_ and 95% air. Cytotoxicity assessment used flow cytometry analysis was performed using splenocytes as effector cells and CellTrace Violet-stained YAC-1 tumor cells as NK cell-targeted cells, with effector cells:target cells ratio of 20:1. The splenic NK cell cytotoxicity against YAC-1 cells was analyzed using CytoFLEX (Beckman Coulter, Inc.). The data were interpreted using CytExpert software (Beckman Coulter, Inc.) and presented as the mean percentage of CellTrace Violet^+^ 7-aminoactinomycin D^+^ YAC-1 cells.

### RNA Extraction and Real-Time Quantitative Polymerase Chain Reaction (RT-qPCR)

To extract mRNA from spleen tissues and peritoneal macrophages isolated from immunocompromised mice, tissues were homogenized using a TissueLyser II (Qiagen, USA). RNA was extracted from the homogenized tissues and peritoneal macrophages using an AccuPrep Universal RNA extraction kit (Bioneer, Republic of Korea) according to the manufacturer's instructions. Then, 800 ng of total RNA was reverse transcribed with ReverTra Ace qPCR RT Master Mix (Toyobo, Japan). RT-qPCR was performed using SYBR Green PCR Master Mix (Toyobo) on an Applied Biosystems Step One Plus real-time PCR system (USA) in the presence of gene-specific primers.

### Quantification of Immunoglobulin and Cytokine Levels

The concentration of serum immunoglobulin (Ig) G and IgA, and cytokines including IL1β, IL2, IL6, and IFNγ in cell culture supernatants were quantified using commercially available ELISA kits (BD Biosciences, USA) according to the manufacturer’s instructions. Briefly, each well was coated overnight with a capture antibody, washed with washing buffer, and blocked for non-specific binding for 1 h. Then, the prepared standards and supernatants were added to the appropriate wells for 2 h. The detection antibody and horseradish peroxidase were mixed with reagent diluents and added to each well for 1 h. The substrate solution was then added to the appropriate wells for 30 min. After 30 min, the stop solution was added to each well. Absorbance was measured at 450 nm using an Epoch microplate reader (BioTek, USA).

### Statistical Analyses

All data were analyzed using GraphPad Prism version 10.4.1 (GraphPad Software, USA). The statistical significance of the differences between multiple groups was evaluated using one-way analysis of variance, followed by Dunnett's post hoc test. All data are expressed as mean ± standard deviation. A *p*-value < 0.05 was considered to be statistically significant (*p < 0.05, **p < 0.01, and ***p < 0.001).

## Results

### TF-343 Restored the Reductions in Body Weight and Organ Indices Caused by Immunosuppression

**Fig. 1:**
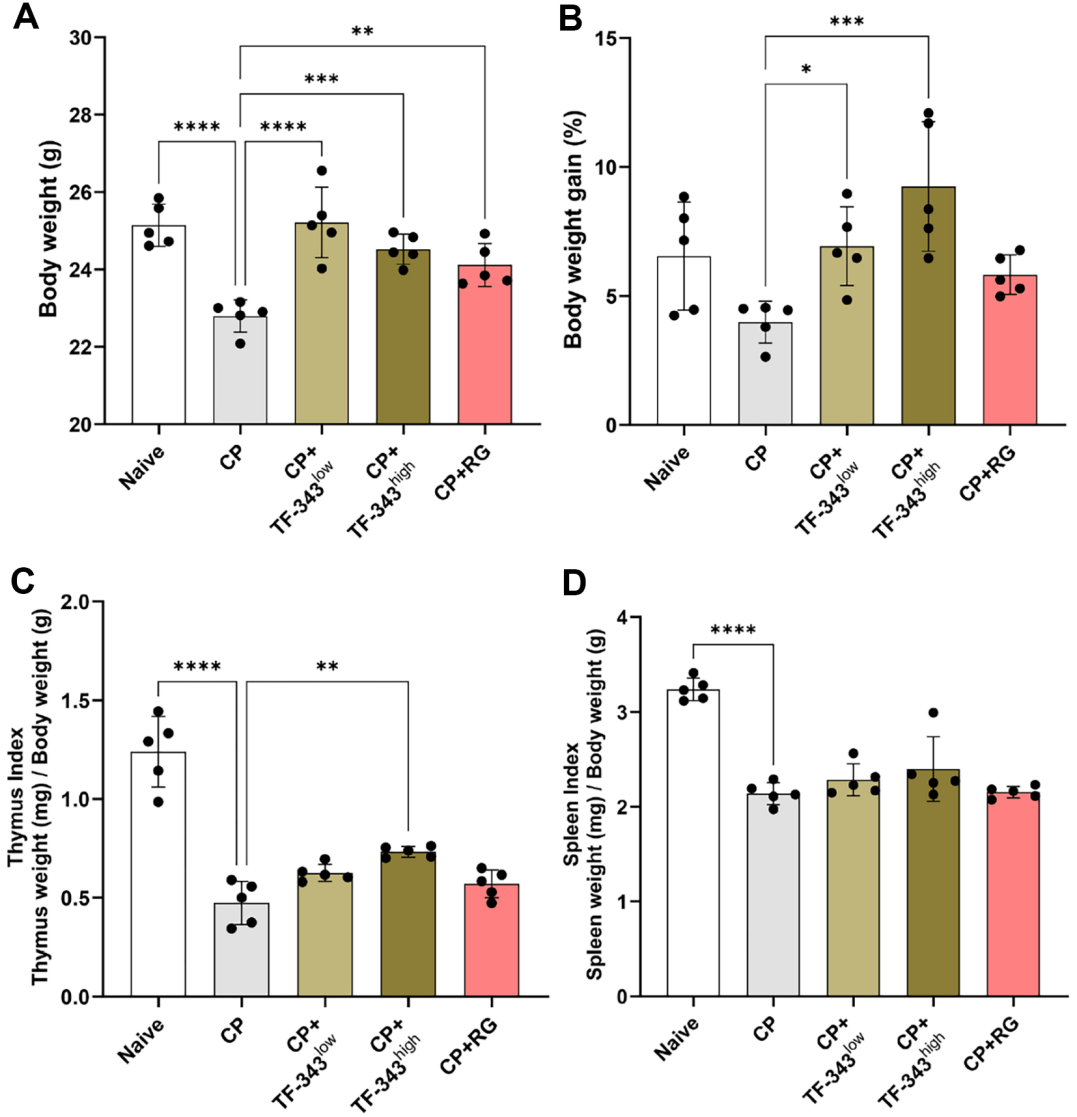
Effect of TF-343 on the body weights and immune organ indices in the CP-induced immunocompromised mice. (**A**) On day 17, mice were euthanized using isoflurane inhalation, and individual body weights were recorded (*n* = 5). (**B**) Weight gain was calculated by subtracting the initial body weight from the final body weight measured on the last day of the experiment (*n* = 5). (**C** and **D**) Thymus (**C**) and spleen (**D**) organ indices were determined by normalizing the weight of each organ (mg) divided by the body weight (g) (*n* = 5). Data are presented as mean ± SD and analyzed using a one-way analysis of variance (*p < 0.05, **p < 0.01, and ***p < 0.001). CP, cyclophosphamide; TF-343, mixed eight medicinal plants hot water extract; TF-343^low^, TF-343 200 mg/kg; TF-343^high^, TF-343 400 mg/kg; RG, red ginseng.

To evaluate the immunomodulatory effects of TF-343, an immunosuppressed mouse model was established using CP injection. The dosage of TF-343 was established based on a previous study and safety assessments, with animals receiving daily oral administration of 200 or 400 mg/kg daily for 17 days [24]. Red ginseng (RG) is widely recognized as an immunomodulator owing to its well-documented and multifaceted immune-enhancing properties [25, 26]. Its bioactive components, particularly ginsenosides and polysaccharides, exert profound effects on both innate and adaptive immunity by activating immune cells via mitogen-activated protein kinase and NFκB signaling, enhancing cytokine production, augmenting natural killer (NK) cell cytotoxicity, and elevating CD4^+^ T cell proportions [27-30]. This evidence shows that RG has been established as a scientifically validated positive control and provides a reliable comparative variable for evaluating the efficacy of new immunomodulators. Thus, we used RG as a positive control to validate the efficacy of TF-343. Body weight on day 17 and body weight gain, which was defined as the percentage change between initial and final body weight, were diminished in the CP group compared with the negative control group. (Fig. 1A and 1B). However, TF-343 administration significantly restored the CP-induced decrease in body weight and body weight gain (Fig. 1A and 1B). The thymus, a primary lymphoid organ, plays a crucial role in T lymphocyte maturation and immune system development through positive and negative selection [31]. The spleen, the largest secondary lymphoid organ in the human body, filters senescent cells, microorganisms, and antigens, thereby contributing to the maintenance of immune homeostasis [32]. Therefore, the relative organ weights of the thymus and spleen, which are normalized to body weight, are widely used as important indicators for assessing the decline or enhancement of immune function [33, 34]. As shown in Fig. 1C and 1D, CP treatment significantly decreased the thymus and spleen indices compared with those in the negative control group. However, TF-343 supplementation resulted in an increase in the immune organ indices compared with the CP group in a concentration-dependent manner (Fig. 1C and 1D). Collectively, our observations demonstrated that TF-343 recovered CP-induced decreases in the thymus, spleen, and body weight in an immunocompromised mouse model.

### TF-343 Compensated Diminished Numbers of Lymphocytes and Leukocytes in Immunocompromised Conditions

**Fig. 2:**
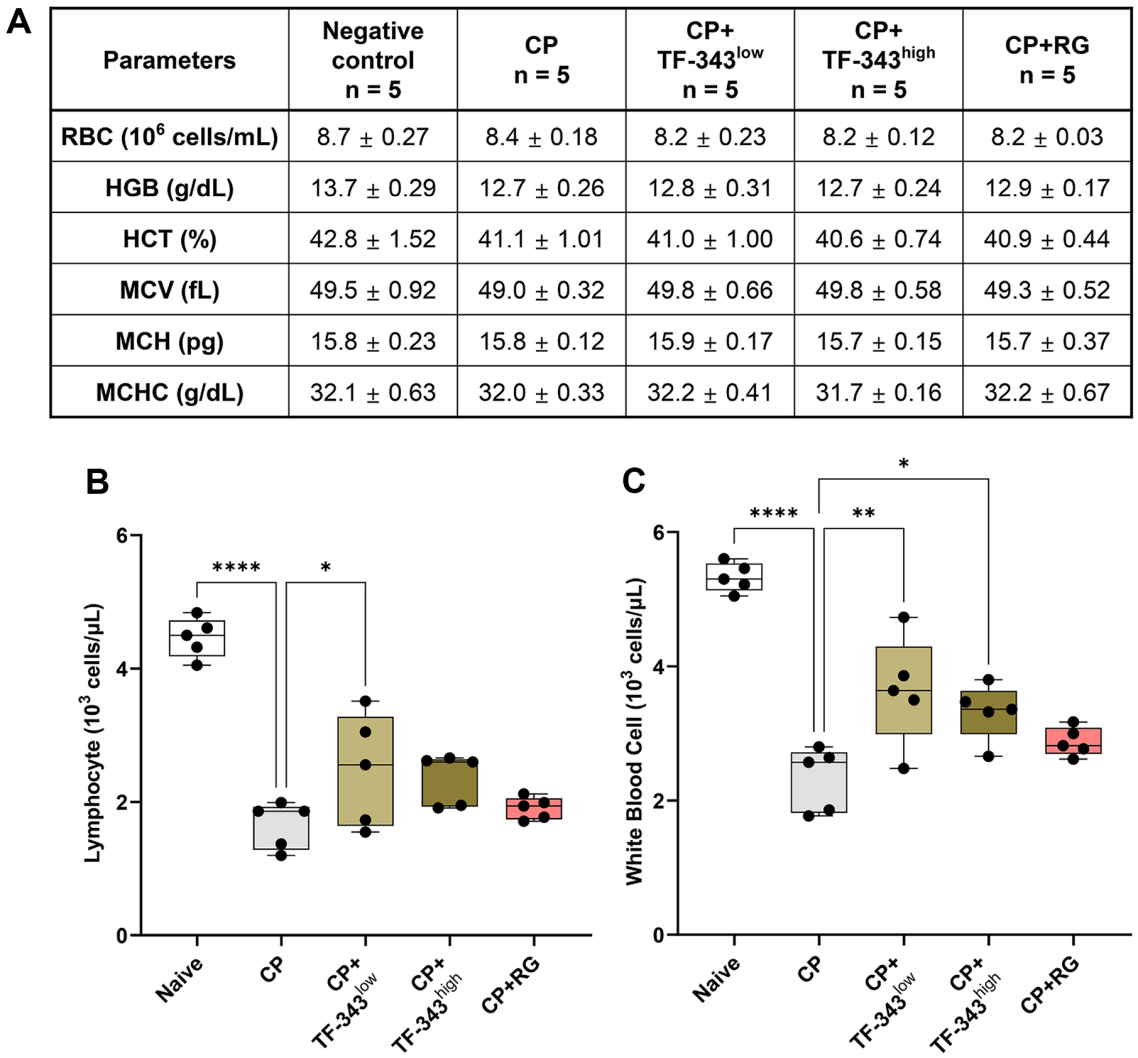
Effect of TF-343 on the hematopoietic compartments of the CP-elicited immunosuppressed mice. (**A-C**) Hematopoietic compartments in the total blood of mice were individually analyzed using an ADVIA 2120i automatic blood cell analyzer (*n* = 5). Data are presented as mean ± SD and analyzed using a one-way analysis of variance (*p < 0.05, **p < 0.01, and ***p < 0.001). CP, cyclophosphamide; TF-343, mixed eight medicinal plants hot water extract; TF-343^low^, TF-343 200 mg/kg; TF-343^high^, TF-343 400 mg/kg; RG, red ginseng.

Next, we examined whether TF-343 affected hematological parameters under immunocompromised conditions. As shown in the hematopoietic cell compartment analysis, red blood cell counts, hemoglobin concentration, and the percentage of red blood cells were slightly decreased by CP treatment (Fig. 2A). Additionally, the mean volume of red blood cells, average amount of hemoglobin in each red blood cell, and mean concentration of hemoglobin in each red blood cell revealed no significant differences in the presence or absence of CP-induced immunosuppression or TF-343 administration (Fig. 2A). The number of lymphocytes and white blood cells was significantly lower in the CP group than in the negative control group (Fig. 2B and 2C). However, TF-343 administration compensated for the diminished lymphocyte and white blood cell counts observed in CP-treated immunocompromised mice (Fig. 2B and 2C). Taken together, our results revealed that TF-343 recovered the decreased numbers of lymphocytes and white blood cells in whole blood of an immunosuppressed mouse model.

### TF-343 Increased Immunoglobulin Production in CP-Induced Immunocompromised Mouse

**Fig. 3:**
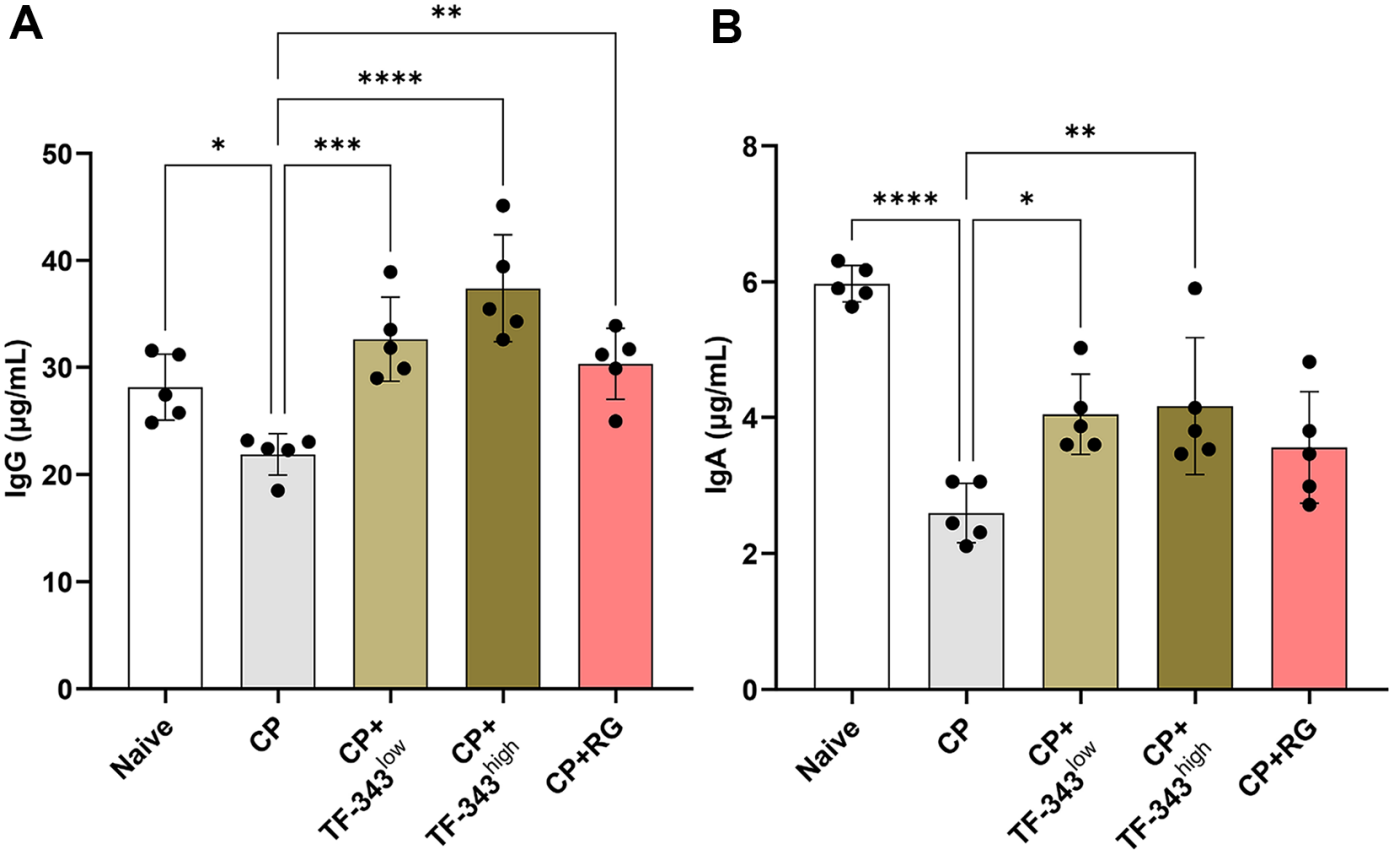
Effect of TF-343 on serum IgG and IgA production in the CP-induced immunocompromised mice. (**A and B**) The serum was isolated from the whole blood of the mouse, and the levels of IgG (**A**) and IgA (**B**) were quantified using an ELISA kit (*n* = 5). Data are presented as mean ± SD and analyzed using a one-way analysis of variance (*p < 0.05, **p < 0.01, and ***p < 0.001). CP, cyclophosphamide; TF-343, mixed eight medicinal plants hot water extract; TF-343^low^, TF-343 200 mg/kg; TF-343^high^, TF-343 400 mg/kg; RG, red ginseng.

Immunoglobulins produced by B lymphocytes play a pivotal role in immune responses by recognizing external antigens, bacteria, and viruses, thereby activating the complement system and inducing antibody-dependent cellular cytotoxicity to neutralize or eliminate antigens [35, 36]. IgG is the most abundant of the five major immunoglobulin classes, accounting for approximately 75% of the total immunoglobulins in the blood, with the remainder distributed in tissue fluids such as lymph and cerebrospinal fluid [37]. IgA, present in small amounts in the blood, can bind to CD89 expressed on innate immune cells and facilitate immune functions, such as phagocytosis and inflammatory responses [38]. Therefore, we investigated whether TF-343 affects immunoglobulin production in whole blood of CP-induced immunosuppressed mice. As shown in Fig. 3A and 3B, the serum levels of IgG and IgA were significantly reduced in the CP group compared with those in the negative control group. The oral administration of TF-343 restored the CP-induced decrease in serum IgG and IgA production (Fig. 3A and 3B). Collectively, our findings showed that TF-343 promoted serum IgG and IgA production in CP-induced immunocompromised mice.

### TF-343 Promoted Splenocyte Proliferation and Splenic NK Cell Cytotoxicity in CP-Induced Immunosuppressed Mice

**Fig. 4:**
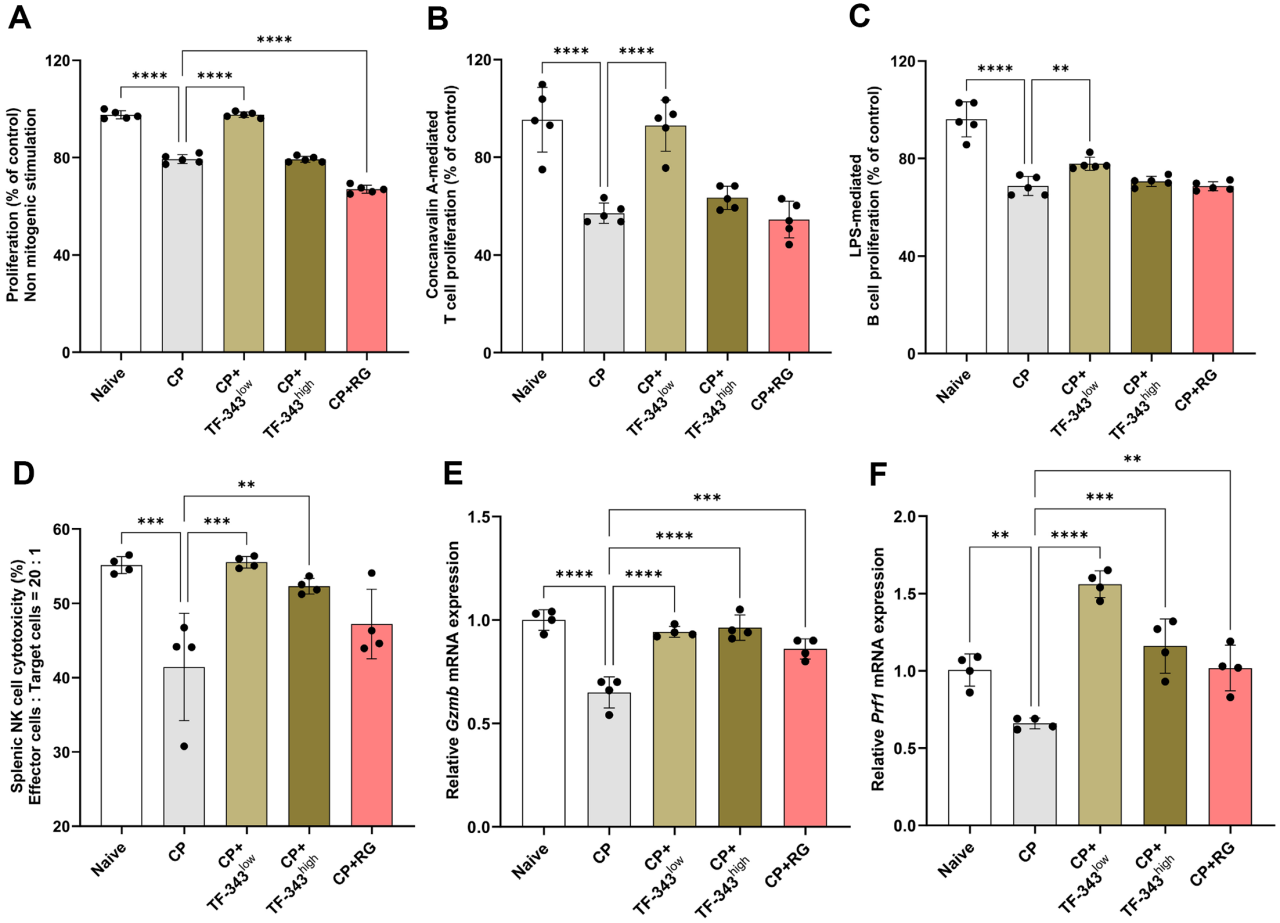
Effect of TF-343 on splenocyte proliferation and splenic NK cell cytotoxicity in the CP-elicited immunosuppressed status. (**A–C**) On day 17, mice were euthanized using isoflurane inhalation; splenocytes were isolated from individual spleens by homogenizing (*n* = 5). The proliferative capacity of total splenocytes in the absence of mitogenic stimulation (**A**) as well as T (**B**) and B (**C**) cell proliferation induced by 1 μg/ml of ConA or LPS, was assessed using the WST-1 assay. (**D**) Splenic NK cell cytotoxicity was analyzed using flow cytometry and represented as the mean percentage of 7- aminoactinomycin D-positive YAC-1 target cells (*n* = 4). (**E** and **F**) Total RNA extracted from the spleen was used to quantify Gzmb (**E**) and Prf1 (**F**) mRNA expression levels using RT‐qPCR (*n* = 4). Data are presented as mean ± SD and analyzed using a one-way analysis of variance (*p < 0.05, **p < 0.01, and ***p < 0.001). CP, cyclophosphamide; TF-343, mixed eight medicinal plants hot water extract; TF-343^low^, TF-343 200 mg/kg; TF-343^high^, TF-343 400 mg/kg; RG, red ginseng.

The spleen is a key lymphoid organ in which various immune cells, such as T and B lymphocytes, macrophages, and neutrophils, are recruited and activated [39]. Proliferation and activation of immune cells in response to antigen exposure are critical indicators of enhanced immune responses. Therefore, we investigated whether TF-343 affected immune cell proliferation in CP-induced immunosuppressed mice. As anticipated, CP treatment significantly decreased splenocyte proliferation compared with the negative control group (Fig. 4A). TF-343 administration rescued the CP-induced decrease in splenocyte proliferation to levels comparable to those of the negative control group (Fig. 4A). Next, to evaluate the effect of TF-343 on T and B lymphocyte proliferation within splenocytes in more detail, we stimulated splenocytes with either concanavalin A or lipopolysaccharide, T cell- and B cell-specific mitogens. CP-induced immunocompromised conditions significantly reduced mitogen-specific T and B lymphocyte proliferation in splenocytes compared with that in the negative control group (Fig. 4B and 4C). However, TF-343 supplementation increased mitogen-specific T and B lymphocyte proliferation (Fig. 4B and 4C). NK cells are major innate immune cells that recognize and induce apoptosis and necrosis in virus-infected cells, abnormal cells, and tumor cells through the secretion of granules, such as perforin and granzymes [40, 41]. In both humans and mice, the spleen has a high proportion of NK cells involved in immune responses, and enhanced NK cytotoxicity is considered an indicator of improved immune function [19, 33, 34]. Thus, we isolated the spleens from mice in each group and used them in *ex vivo* experiments to investigate the effect of TF-343 on NK cell cytotoxicity against YAC-1 tumor cells. The CP-induced immunosuppressive status exhibited significantly impaired NK cytotoxicity (41.43%) against YAC-1 target cells at an effector-to-target ratio of 20:1 compared with that in the negative control group (55.14%). (Fig. 4D). However, oral administration of TF-343 at 200 mg/kg (55.03%) and 400 mg/kg (52.30%) significantly restored impaired NK cell cytotoxicity after CP treatment (Fig. 4D). Accordingly, we investigated whether TF-343 enhanced the expression of NK cell function-related genes in CP-induced immunosuppressed mice. Consistent with the splenic NK cell cytotoxicity analysis, supplementation with TF-343 significantly restored the decrease in *Gzmb* and *Prf1* mRNA expression under CP-induced immunocompromised conditions. Taken together, our findings demonstrate that TF-343 enhances the proliferation of splenocytes, including T and B lymphocytes, as well as the cytotoxicity and granule production in splenic NK cells.

### TF-343 Enhanced mRNA and Protein Expression of IL2 and IFNγ in Splenocytes under Immunocompromised Status

**Fig. 5:**
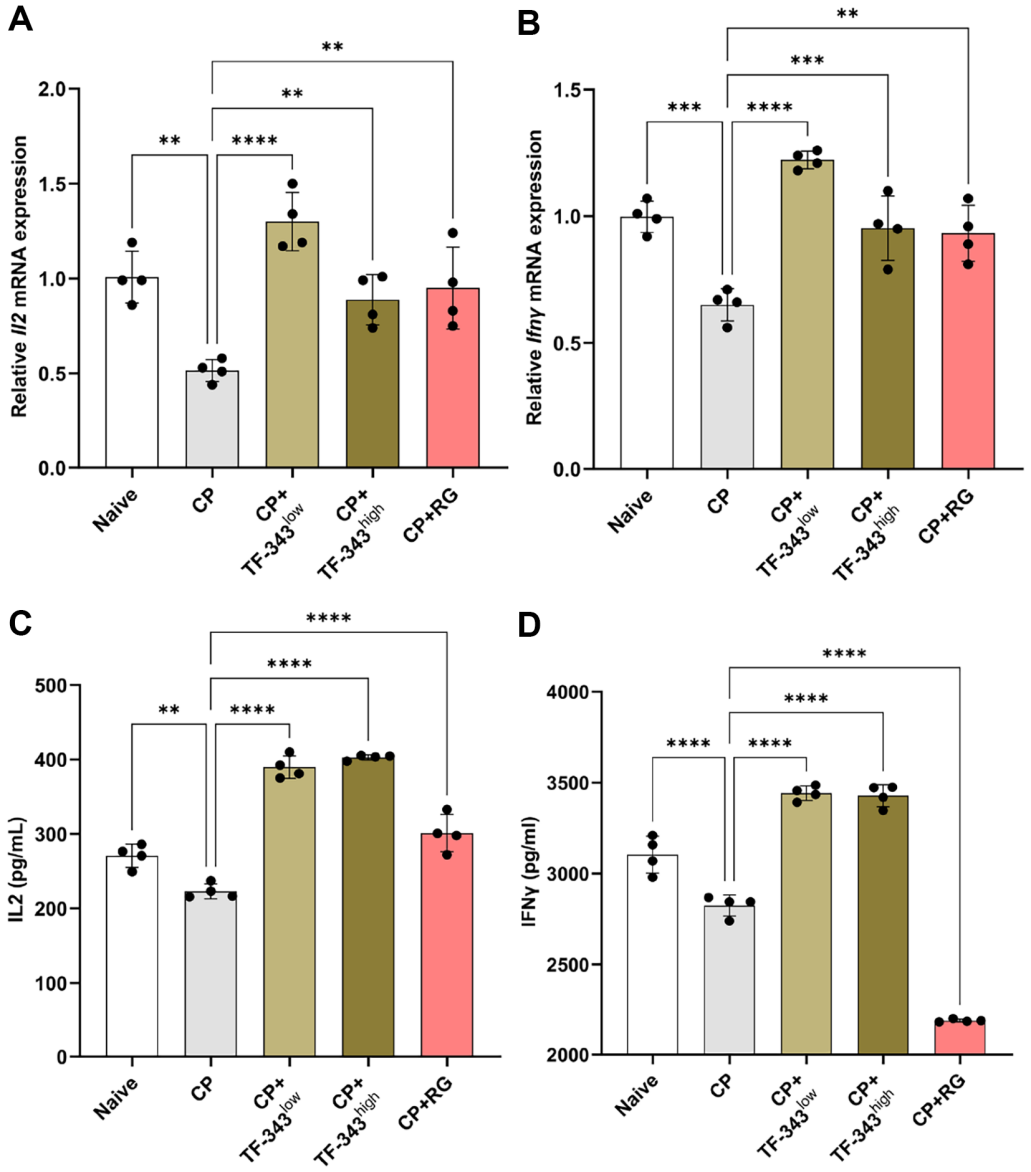
Effect of TF-343 on splenocyte IL2 and IFNγ production in the CP-induced immunocompromised condition. (**A–D**) On day 17, mice were sacrificed under inhalation anesthesia, and spleens were harvested. Splenocytes were isolated and cultured for 24 and 48 h with a complete medium. The expression of IL2 and IFNγ mRNA (**A** and **B**) and protein (**C** and **D**) levels were quantified using RT-qPCR and ELISA (*n* = 4). Data are presented as mean ± SD and analyzed using a oneway analysis of variance (*p < 0.05, **p < 0.01, and ***p < 0.001). CP, cyclophosphamide; TF-343, mixed eight medicinal plants hot water extract; TF-343^low^, TF-343 200 mg/kg; TF-343^high^, TF-343 400 mg/kg; RG, red ginseng.

IL2 and IFNγ are pivotal immunostimulatory and immunomodulatory factors that play important roles in T cell differentiation, proliferation, and activation, thereby contributing to host defense and immune homeostasis [8, 42]. To investigate the effect of TF-343 on the expression of immune response-associated factors, we performed *ex vivo* experiments using splenocytes isolated from the spleens of the mice in each experimental group. As shown in Fig. 5A and 5B, CP-induced immunocompromised status decreased *Il2* and *Ifnγ* mRNA expression in splenocytes compared with that in the negative control group. However, oral administration of TF-343 significantly restored *Il2* and *Ifnγ* mRNA expression compared with levels in the CP group (Fig. 5A and 5B). Consistent with these observations, CP-induced impaired IL2 and IFNγ production in splenocytes were significantly recovered by TF-343 supplementation (Fig. 5C and 5D). Consequently, our observations showed that TF-343 promoted IL2 and IFNγ mRNA and protein expression in splenocytes under immunocompromised conditions.

### TF-343 Promoted Gene Expressions associated with Phagocytosis and Immune Responses in Peritoneal Macrophages from CP-Induced Immunosuppressed Mice

**Fig. 6:**
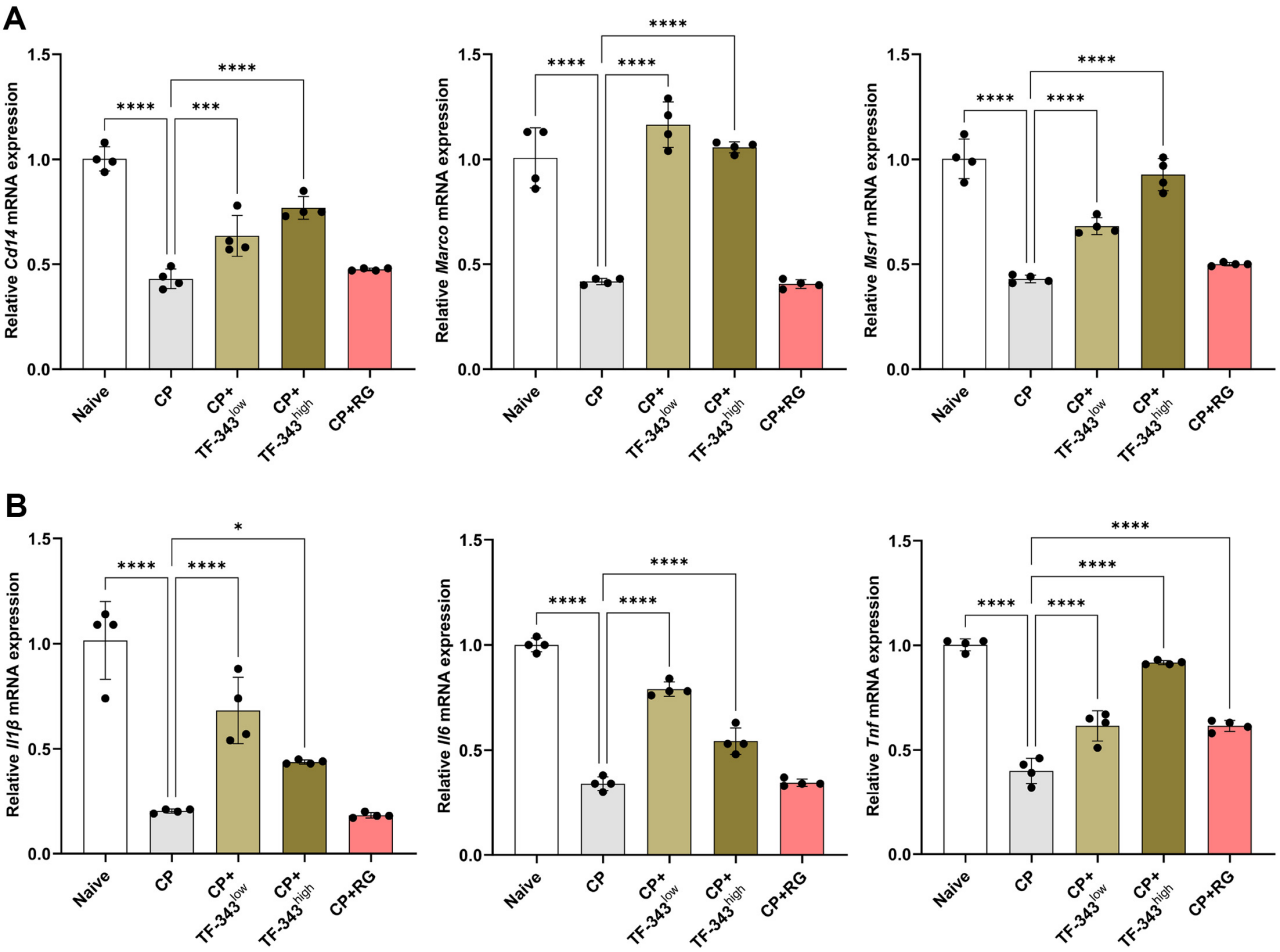
Effect of TF-343 on gene expression associated with macrophage functions in the CP-elicited immunosuppressed condition. (**A** and **B**) Total RNA extracted from the spleen of each mouse was used to quantify *Cd14*, *Marco*, *Msr1* (**A**) *Il1β*, *Il6*, and *Tnf* mRNA expression levels (**B**) using RT‐qPCR (*n* = 4). Data are presented as mean ± SD and analyzed using a one-way analysis of variance (*p < 0.05, **p < 0.01, and ***p < 0.001). CP, cyclophosphamide; TF-343, mixed eight medicinal plants hot water extract; TF-343^low^, TF-343 200 mg/kg; TF-343^high^, TF-343 400 mg/kg; RG, red ginseng.

Macrophages are pivotal components of the innate immune system that play critical roles in the initiation and orchestration of immune responses [4]. Macrophages directly eliminate invading pathogens, microorganisms, and damaged cells through phagocytosis and the presentation of antigens to T lymphocytes, resulting in a bridge between innate and adaptive immunity, thereby shaping subsequent immunological outcomes [43]. To investigate the effects of TF-343 on macrophage function, peritoneal macrophages were isolated from immunocompromised mice, and *ex vivo* experiments were conducted. The expression of genes associated with phagocytosis and immune responses was assessed using RT-qPCR. The mRNA expression of macrophage phagocytic markers, including *Cd14*, *Marco*, and *Msr1*, was markedly decreased in the CP group compared with that in the negative control group (Fig. 6A). However, oral administration of TF-343 reversed the decrease in *Cd14*, *Marco*, and *Msr1* mRNA expression under CP-induced immunocompromised conditions (Fig. 6A). Macrophages are the major source of pro-inflammatory cytokines such as IL1β, IL6, and TNF that play central roles in infection, inflammation, and immune responses [43]. These cytokines contribute to the regulation of cell death as well as the recruitment and activation of immune cells [10]. Thus, we investigated whether TF-343 promoted *Il1β*, *Il6*, and *Tnf* mRNA expression in macrophages under CP-elicited immunosuppressed conditions. As anticipated, supplementation with TF-343 significantly restored decreased *Il1β*, *Il6*, and *Tnf* mRNA expression under CP-induced immunocompromised conditions (Fig. 6B). Collectively, our results demonstrate that TF-343 increases phagocytosis- and immune response-associated gene expression in immunocompromised macrophages.[Table T1]

## Discussion

Our findings showed that supplementation with the multi-herbal hot water extract of TF-343 promoted immune function in a CP-induced immunocompromised mouse model. The key findings of this study are as follows. TF-343 effectively restored CP-induced reductions in body weight and immune organ indices, including those of the thymus and spleen. Under CP-induced immunosuppressive conditions, TF-343 compensated for the decreased numbers of lymphocytes and leukocytes in whole blood. Moreover, TF-343 significantly elevated serum levels of IgG and IgA in immunocompromised mice. TF-343 rescued the impaired proliferation of splenocytes, including T and B lymphocytes, and enhanced NK cell cytotoxicity. In parallel, TF-343 upregulated mRNA expression of the major cytolytic mediators *Gzmb* and *Prf1*. Additionally, TF-343 promoted both mRNA and protein expression of IL2 and IFNγ in the spleen, suggesting potentiation of T cell-mediated immunity. Importantly, TF-343 restored the mRNA expression that was associated with phagocytic activity (*Cd14*, *Marco*, and *Msr1*) and immune response (*Il1β*, *Il6*, and *Tnf*) of macrophages under CP-induced immunocompromised status. Collectively, these observations demonstrate that TF-343 effectively reverses CP-induced immune suppression by enhancing both innate and adaptive immune responses. Our findings suggest that TF-343 is a promising immunomodulatory agent for the prevention and management of compromised immune function.

An immunocompromised status refers to a condition in which the immune system is unable to function properly owing to congenital or acquired factors, resulting in heightened susceptibility to infections and an increased risk for the onset and progression of malignancies and autoimmune disorders [44]. The major causes of immunosuppression include radiotherapy, chemotherapy, immunosuppressive therapy following organ transplantation, human immunodeficiency virus infection, malnutrition, chronic diseases, and advanced age [44, 45]. A compromised immune system is marked by impaired function of immune cells, such as leukocytes and NK cells, with a reduced capacity to recognize and eliminate pathogens [46-48]. Consequently, even low-virulence microbes can cause severe and potentially life-threatening infections [49]. Consistent with these observations, our study demonstrated that CP-induced immunosuppression in mice resulted in a marked decrease in immune organ indices, reduced counts of peripheral blood lymphocytes and leukocytes, and impaired the functional activity of NK cells, splenocytes, and macrophages. Recent clinical studies have shown that immunocompromised individuals are at markedly higher risk of severe respiratory infections, with significantly elevated morbidity and mortality rates [50, 51]. In line with this, a cohort study involving 1,491 patients admitted to intensive care units for coronavirus disease 19 showed that approximately 30% were immunocompromised and that these individuals exhibited significantly higher intensive care unit mortality than non-immunocompromised patients [52]. Mumford *et al*. demonstrated that immunosuppressed populations tend to exhibit reduced vaccine immunogenicity and efficacy, with a higher likelihood of generating undetectable immune responses than healthy controls. Moreover, persistent and worsening coronavirus disease 19 infections in immunocompromised hosts are associated with enhanced viral evolution and the emergence of novel variants [53]. However, emerging evidence supports the promise of nutritional immunology to enhance vaccine response and immune resilience via microbiota modulation and immunonutrition [54, 55]. These observations underscore the demand for tailored, multifaceted strategies to prevent or mitigate immunosuppression, including immunomodulatory supplements, probiotics to modulate gut immunity, optimized vaccination protocols, targeted nutritional interventions, and individualized physical activity regimens. Moreover, recent studies demonstrated the use of multi-herbal formulations that integrate distinct bioactivities, such as immunomodulation, anti-inflammatory effects, and the promotion of beneficial gut commensals, as a comprehensive strategy to sustain immune homeostasis [56, 57]. These combinatorial approaches are increasingly recognized for their potential in reducing susceptibility to respiratory infections and in contributing to the broader prevention and management of diverse pathological conditions.

Herbal medicines traditionally used in East Asian medicine have been shown to modulate a range of physiological processes, including inflammatory responses, oxidative stress, and immune responses [58]. Botanical therapeutics are generally considered to have a more favorable safety profile than synthetic pharmaceuticals, with lower risks of adverse effects and drug resistance [22]. Traditional herbal formulations are often developed based on rigorously established combinatorial principles that emphasize the synergistic interactions among multiple components. This polyherbal approach enhances the overall bioactivity and mitigates the toxicity or side effects associated with the individual constituents [21, 23]. TF-343 is a multicomponent hot water extract derived from eight medicinal herbs and is known for its anti-inflammatory and antioxidant properties [24]. Among these eight constituents, *G. max* [59], *G. uralensis* [60], *L. japonica* [61], and *T. platycarpum* [62] have recently shown immunomodulatory effects in various *in vitro* and *in vivo* studies. In particular, extracts of *G. uralensis* have been demonstrated to modulate immune function by attenuating intestinal inflammation through regulation of the NOD2/RIP2/NFκB signaling cascade [60]. Similarly, polysaccharides isolated from *T. platycarpum* roots were shown to activate macrophages via toll-like receptor (TLR) 2, TLR4, and complement receptor 3, leading to enhanced production of nitric oxide and cytokines through stimulation of the mitogen-activated protein kinase and NFκB pathways [62]. These findings suggest that TF-343 may serve as a promising immune enhancer by leveraging the synergistic pharmacological activities of its constituent herbs. However, the pharmacological mechanisms of herbal mixtures are inherently complex and cannot be reduced to a simple sum of their components. This underscores the need for system-level approaches to fully elucidate their immunological and therapeutic potential. Further studies are needed to identify the active substances and their physiological activities and associated mechanisms in TF-343 using ultra-high-performance liquid chromatography with quadrupole time-of-flight mass spectrometry. Furthermore, in the context of multi-herbal formulations, it is well recognized that biological efficacy often reaches a plateau beyond a certain threshold dose, rather than exhibiting a linear dose–response relationship. Accordingly, our present findings underscore the necessity of future investigations to determine whether the immunomodulatory activity of TF-343 can be achieved at lower dose ranges.

## Figures and Tables

**Table 1 T1:** List of primer sequences used in RT‐qPCR analysis.

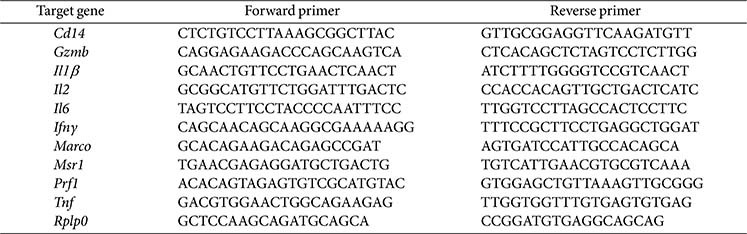

## References

[1] Gasteiger G, Rudensky AY (2014). Interactions between innate and adaptive lymphocytes. Nat. Rev. Immunol..

[2] Hoebe K, Janssen E, Beutler B (2004). The interface between innate and adaptive immunity. Nat. Immunol..

[3] Wang R, Jaw JJ, Stutzman NC, Zou Z, Sun PD (2012). Natural killer cell-produced IFN-gamma and TNF-alpha induce target cell cytolysis through up-regulation of ICAM-1. J. Leukoc. Biol..

[4] Murray PJ, Wynn TA (2011). Protective and pathogenic functions of macrophage subsets. Nat. Rev. Immunol..

[5] Iwasaki A, Medzhitov R (2015). Control of adaptive immunity by the innate immune system. Nat. Immunol..

[6] Dorner T, Radbruch A (2007). Antibodies and B cell memory in viral immunity. Immunity.

[7] Slifka MK, Whitton JL (2000). Antigen-specific regulation of T cell-mediated cytokine production. Immunity.

[8] Pol JG, Caudana P, Paillet J, Piaggio E, Kroemer G (2020). Effects of interleukin-2 in immunostimulation and immunosuppression. J. Exp. Med..

[9] Tanaka T, Narazaki M, Kishimoto T (2014). Il-6 in inflammation, immunity, and disease. Cold Spring Harb. Perspect. Biol..

[10] McHugh S, Deighton J, Rifkin I, Ewan P (1996). Kinetics and functional implications of Th1 and Th2 cytokine production following activation of peripheral blood mononuclear cells in primary culture. Eur. J. Immunol..

[11] Emadi A, Jones RJ, Brodsky RA (2009). Cyclophosphamide and cancer: golden anniversary. Nat. Rev. Clin. Oncol..

[12] Zhou X, Dong Q, Kan X, Peng L, Xu X, Fang Y, Yang J (2018). Immunomodulatory activity of a novel polysaccharide from *Lonicera Japonica* in immunosuppressed mice induced by cyclophosphamide. PLoS One.

[13] Dobson J (2014). Reducing the side effects of cyclophosphamide chemotherapy in dogs. Vet. Rec..

[14] Ahlmann M, Hempel G (2016). The effect of cyclophosphamide on the immune system: implications for clinical cancer therapy. Cancer Chemother. Pharmacol..

[15] Brode S, Cooke A (2008). Immune-potentiating effects of the chemotherapeutic drug cyclophosphamide. Crit. Rev. Immunol..

[16] Sistigu A, Viaud S, Chaput N, Bracci L, Proietti E, Zitvogel L (2011). Immunomodulatory effects of cyclophosphamide and implementations for vaccine design. Semin. Immunopathol..

[17] Shyam Sunder S, Sharma UC, Pokharel S (2023). Adverse effects of tyrosine kinase inhibitors in cancer therapy: pathophysiology, mechanisms and clinical management. Signal Transduct. Target. Ther..

[18] de Jonge ME, Huitema AD, Rodenhuis S, Beijnen JH (2005). Clinical pharmacokinetics of cyclophosphamide. Clin. Pharmacokinet..

[19] Cho CH, Youm G, Lim KM, Kim M, Lee DK, Cho YB (2025). Immune-enhancing effects of enzymatic hydrolysates of peanut sprouts in Raw 264.7 macrophages and cyclophosphamide-induced immunosuppressed mouse model. Food Res. Int..

[20] Mughal KS, Ikram M, Uddin Z, Rashid A, Rashid U, Khan M (2024). Syringic acid improves cyclophosphamide-induced immunosuppression in a mouse model. Biochem. Biophys. Res. Commun..

[21] Jang SY, Song HA, Park MJ, Chung KS, Lee JK, Jang EY (2025). Immunomodulatory effects of a standardized botanical mixture comprising *Angelica gigas* roots and *Pueraria lobata* flowers through the TLR^2^/6 pathway in RAW 264.7 macrophages and cyclophosphamide-induced immunosuppression mice. Pharmaceuticals (Basel).

[22] Rodriguez-Mateos A, Cifuentes-Gomez T, George TW, Spencer JP (2014). Impact of cooking, proving, and baking on the (poly)phenol content of wild blueberry. J. Agric. Food Chem..

[23] Zhou X, Seto SW, Chang D, Kiat H, Razmovski-Naumovski V, Chan K (2016). Synergistic effects of chinese herbal medicine: a comprehensive review of methodology and current research. Front. Pharmacol..

[24] Kim DI, Song MK, Kim SH, Park CY, Lee K (2019). TF-343 Alleviates diesel exhaust particulate-induced lung inflammation via modulation of nuclear factor-kappaB signaling. J. Immunol. Res..

[25] Kang S, Min H (2012). Ginseng, the 'immunity boost': the effects of panax ginseng on immune system. J. Ginseng Res..

[26] Hyun SH, Ahn HY, Kim HJ, Kim SW, So SH, In G (2021). Immuno-enhancement effects of korean red ginseng in healthy adults: a randomized, double-blind, placebo-controlled trial. J. Ginseng Res..

[27] Cho YJ, Son HJ, Kim KS (2014). A 14-week randomized, placebo-controlled, double-blind clinical trial to evaluate the efficacy and safety of ginseng polysaccharide (Y-75). J. Transl. Med..

[28] Kim IK, Lee KY, Kang J, Park JS, Jeong J (2021). Immune-modulating effect of korean red ginseng by balancing the ratio of peripheral T lymphocytes in bile duct or pancreatic cancer patients with adjuvant chemotherapy. In Vivo.

[29] Kim JY, Germolec DR, Luster MI (1990). Panax ginseng as a potential immunomodulator: studies in mice. Immunopharmacol. Immunotoxicol..

[30] Ratan ZA, Youn SH, Kwak YS, Han CK, Haidere MF, Kim JK (2021). Adaptogenic effects of panax ginseng on modulation of immune functions. J. Ginseng Res..

[31] Ladi E, Yin X, Chtanova T, Robey EA (2006). Thymic microenvironments for T cell differentiation and selection. Nat. Immunol..

[32] Lewis SM, Williams A, Eisenbarth SC (2019). Structure and function of the immune system in the spleen. Sci. Immunol..

[33] Zhang WN, Gong LL, Liu Y, Zhou ZB, Wan CX, Xu JJ (2020). Immunoenhancement effect of crude polysaccharides of *Helvella leucopus* on cyclophosphamide-induced immunosuppressive mice. J. Funct. Foods.

[34] Kim GD, Yoo SH, Song JH, Lim KM, Lim EY, Yoo JY (2024). *Nypa fruticans* Wurmb extract recovered compromised immune status induced by forced swimming in a mouse model. J. Microbiol. Biotechnol..

[35] Meffre E, Casellas R, Nussenzweig MC (2000). Antibody regulation of B cell development. Nat. Immunol..

[36] Carroll MC, Isenman DE (2012). Regulation of humoral immunity by complement. Immunity.

[37] Nimmerjahn F, Vidarsson G, Cragg MS (2023). Effect of posttranslational modifications and subclass on IgG activity: from immunity to immunotherapy. Nat. Immunol..

[38] Mantis NJ, Rol N, Corthesy B (2011). Secretory IgA's complex roles in immunity and mucosal homeostasis in the gut. Mucosal Immunol..

[39] Bronte V, Pittet MJ (2013). The spleen in local and systemic regulation of immunity. Immunity.

[40] Vivier E, Tomasello E, Baratin M, Walzer T, Ugolini S (2008). Functions of natural killer cells. Nat. Immunol..

[41] Suzui M, Kawai T, Kimura H, Takeda K, Yagita H, Okumura K (1985). Natural killer cell lytic activity and CD56(dim) and CD56(bright) cell distributions during and after intensive training. J. Appl. Physiol..

[42] Billiau A, Heremans H, Vermeire K, Matthys P (1998). Immunomodulatory properties of interferon-gamma. an update. Ann. N Y Acad. Sci..

[43] Murray PJ, Allen JE, Biswas SK, Fisher EA, Gilroy DW, Goerdt S, et al. Macrophage activation and polarization: nomenclature and experimental guidelines. *Immunity* **41:** 14-20. 10.1016/j.immuni.2014.06.008 25035950 PMC4123412

[44] Dropulic LK, Lederman HM. 2016. Overview of infections in the immunocompromised host. *Microb. Spectr.* **4**.10.1128/microbiolspec.DMIH2-0026-2016. 10.1128/microbiolspec.DMIH2-0026-2016 27726779 PMC8428766

[45] Otani IM, Lehman HK, Jongco AM, Tsao LR, Azar AE, Tarrant TK (2022). Practical guidance for the diagnosis and management of secondary hypogammaglobulinemia: a work group report of the AAAAI primary immunodeficiency and altered immune response committees. J. Allergy Clin. Immunol..

[46] Barnes E, Goodyear CS, Willicombe M, Gaskell C, Siebert S, de Silva TI (2023). SARS-CoV-2-specific immune responses and clinical outcomes after COVID-19 vaccination in patients with immune-suppressive disease. Nat. Med..

[47] Chen S, Zhu H, Jounaidi Y (2024). Comprehensive snapshots of natural killer cells functions, signaling, molecular mechanisms and clinical utilization. Signal Transduct. Target. Ther..

[48] Franquet T (2004). Respiratory infection in the aids and immunocompromised patient. Eur. Radiol..

[49] Soni J, Sinha S, Pandey R (2024). Understanding bacterial pathogenicity: a closer look at the journey of harmful microbes. Front. Microbiol..

[50] DeWolf S, Laracy JC, Perales MA, Kamboj M, van den Brink MRM, Vardhana S (2022). SARS-CoV-2 in immunocompromised individuals. Immunity.

[51] Bytyci J, Ying Y, Lee LYW (2024). Immunocompromised individuals are at increased risk of COVID-19 breakthrough infection, hospitalization, and death in the post-vaccination era: a systematic review. Immun. Inflamm. Dis..

[52] Pander J, Termorshuizen F, de Lange DW, Beekman-Hendriks W, Lanfermeijer J, Bakhshi-Raiez F (2025). The impact of the COVID-19 omicron variant on immunocompromised patients: ICU admissions and increased mortality. Infect. Dis. Ther..

[53] Mumford L, Hogg R, Taylor A, Lanyon P, Bythell M, McPhail S (2025). Impact of SARS-CoV-2 spike antibody positivity on infection and hospitalisation rates in immunosuppressed populations during the omicron period: the MELODY study. Lancet.

[54] Di Renzo L, Franza L, Monsignore D, Esposito E, Rio P, Gasbarrini A (2022). Vaccines, microbiota and immunonutrition: food for thought. Vaccines (Basel).

[55] Mazziotta C, Tognon M, Martini F, Torreggiani E, Rotondo JC (2023). Probiotics mechanism of action on immune cells and beneficial effects on human health. Cells.

[56] Santamarina AB, de Freitas JA, Franco LAM, Nehmi-Filho V, Fonseca JV, Martins RC (2024). Nutraceutical blends predict enhanced health via microbiota reshaping improving cytokines and life quality: a brazilian double-blind randomized trial. Sci. Rep..

[57] Medina-Larque AS, Rodriguez-Daza MC, Roquim M, Dudonne S, Pilon G, Levy E (2022). Cranberry polyphenols and agave agavins impact gut immune response and microbiota composition while improving gut barrier function, inflammation, and glucose metabolism in mice fed an obesogenic diet. Front. Immunol..

[58] Pan MH, Chiou YS, Tsai ML, Ho CT (2011). Anti-inflammatory activity of traditional Chinese medicinal herbs. J. Tradit. Complement. Med..

[59] Wen L, Jiang Y, Zhou X, Bi H, Yang B (2021). Structure identification of soybean peptides and their immunomodulatory activity. Food Chem..

[60] Zhong R, Wen C, Qiu Y, Shen X, Sun Z, Peng L (2025). Anti-inflammatory and immunomodulatory effects of *Glycyrrhiza uralensis* fisch. On ulcerative colitis in rats: role of nucleotide-binding oligomerization domain 2/receptor-interacting protein 2/nuclear factor-kappa B signaling pathway. J. Ethnopharmacol..

[61] Zhang T, Liu H, Ma P, Huang J, Bai X, Liu P (2022). Immunomodulatory effect of polysaccharides isolated from *Lonicera Japonica* Thunb. in cyclophosphamide-treated BALB/c mice. Heliyon.

[62] Talapphet N, Palanisamy S, Li C, Ma N, Prabhu NM, You S (2021). Polysaccharide extracted from *Taraxacum platycarpum* root exerts immunomodulatory activity via MAPK and NF-KappaB pathways in Raw264.7 cells. J. Ethnopharmacol..

